# Gait-Related Brain Activation During Motor Imagery of Complex and Simple Ambulation in Parkinson's Disease With Freezing of Gait

**DOI:** 10.3389/fnagi.2021.731332

**Published:** 2021-09-22

**Authors:** Hui-Chun Huang, Chun-Ming Chen, Ming-Kuei Lu, Bey-Ling Liu, Chia-Ing Li, Jui-Cheng Chen, Guei-Jane Wang, Hsiu-Chen Lin, Jeng-Ren Duann, Chon-Haw Tsai

**Affiliations:** ^1^Graduate Institute of Clinical Medical Science, China Medical University, Taichung, Taiwan; ^2^Division of Parkinson's Disease and Movement Disorders, Department of Neurology, China Medical University Hospital, Taichung, Taiwan; ^3^Neuroscience Laboratory, Department of Neurology, China Medical University Hospital, Taichung, Taiwan; ^4^School of Medicine, College of Medicine, China Medical University, Taichung, Taiwan; ^5^Graduate Institute of Biomedical Sciences, China Medical University, Taichung, Taiwan; ^6^Department of Medical Imaging, China Medical University Hospital, Taichung, Taiwan; ^7^Neuroscience and Brain Disease Center, College of Medicine, China Medical University, Taichung, Taiwan; ^8^Department of Medical Research, China Medical University Hospital, Taichung, Taiwan; ^9^Department of Neurology, China Medical University Hsinchu Hospital, Hsinchu, Taiwan; ^10^Department of Health and Nutrition Biotechnology, Asia University, Taichung, Taiwan; ^11^Department of Physical Therapy, China Medical University, Taichung, Taiwan; ^12^Institute of Education, National Yang Ming Chiao Tung University, Hsinchu, Taiwan; ^13^Institute for Neural Computation, University of California, San Diego, La Jolla, CA, United States

**Keywords:** Parkinson's disease, freezing of gait, motor imagery, functional magnetic resonance imaging, gait disorders

## Abstract

**Background:** Freezing of gait (FOG) in Parkinson's disease (PD) is a devastating clinical phenomenon that has a detrimental impact on patients. It tends to be triggered more often during turning (complex) than during forwarding straight (simple) walking. The neural mechanism underlying this phenomenon remains unclear and requires further elucidation.

**Objective:** To investigate the differences in cerebral functional magnetic resonance imaging responses between PD patients with and without FOG during explicitly video-guided motor imagery (MI) of various complex (normal, freezing) and simple (normal, freezing) walking conditions.

**Methods:** We recruited 34 PD patients, namely, 20 with FOG and 14 without FOG, and 15 normal controls. Participants underwent video-guided MI of turning and straight walking, with and without freezing, while their brain blood oxygen level-dependent (BOLD) activities were measured. Gait analysis was performed.

**Results:** While comparing FOG turning with FOG straight walking, freezers showed higher activation of the superior occipital gyrus, left precentral gyrus, and right postcentral gyrus compared with non-freezers. Normal controls also manifest similar findings compared with non-freezers, except no difference was noted in occipital gyrus activity between the two groups. Freezers also displayed a higher effect size in the locomotor regions than non-freezers during imagery of normal turning.

**Conclusions:** Our findings suggest that freezers require a higher drive of cortical and locomotion regions to overcome the overinhibition of the pathways in freezers than in non-freezers. Compared with simple walking, increased dorsal visual pathway and deep locomotion region activities might play pivotal roles in tackling FOG in freezers during complex walking.

## Introduction

Freezing of gait (FOG) is manifested with “brief, episodic absence of, or marked reduction of forward progression of the feet despite the intention to walk” (Nutt et al., 2011). It has been identified to be a major cause of falls in patients with Parkinson's disease (PD) (Nonnekes et al., 2015). Gait can be either simple (i.e., forward straight walking) or complex (i.e., turning), and FOG tended to be elicited more during complex walking situations (Rahman et al., 2008a; Spildooren et al., 2010; Shine et al., 2012; Smulders et al., 2016; Zhang et al., 2016). During turning, the left and right legs step asymmetrically, and the center of mass temporarily shifts laterally, causing body instability (Bengevoord et al., 2016). Therefore, turning, which requires more complex neural control than straight walking, is often more likely to cause freezing in PD patients with FOG (PD_FOG_). From the pharmacological perspective, the fact that dopaminergic treatment might benefit straight walking more than turning in PD patients (Smulders et al., 2016) suggests different pathophysiologies underlying the two ambulatory situations. Although different gait kinematics in PD_FOG_ during turning have been postulated (Bertoli et al., 2019; Mitchell et al., 2019), the neurophysiological evidence at the cerebral level for FOG in these various scenarios remains pending and requires further investigation.

The current findings of the higher neural level (cerebellum, basal ganglia, locomotion regions, and cortex) activities of FOG mainly arise from the resting-state functional magnetic resonance imaging (fMRI) studies (Nutt, 2013; Gilat et al., 2015; Wang et al., 2016; Bharti et al., 2019; Potvin-Desrochers et al., 2019). However, FOG is a dynamic disorder that tended to be developed during motion. In this regard, it is difficult to reflect the neural activities by resting fMRI. To overcome such obstacle, the motor imagery (MI) method would be suitable for investigating the conditions relevant to FOG. MI has been defined as the conscious mental simulation of actions involving our brain's motor representations in a way that is similar to when we perform actual movements (Jeannerod and Decety, 1995). It can usually be achieved by purely imagining the motion or by a combo of “explicit guidance plus imagery” (Szameitat et al., 2007; La Fougere et al., 2010; Hetu et al., 2013; Duann and Chiou, 2016). MI activates the brain's mirror neurons and motor-related brain areas, including the frontal–parietal network, subcortical, cerebellar, and primary motor cortex similar to motor execution (Szameitat et al., 2007; La Fougere et al., 2010; Hetu et al., 2013; Duann and Chiou, 2016). By MI and the region of interest (ROI) in fMRI, Snijders et al. revealed the elevated activation in the mesencephalic locomotion region (MLR) in PD_FOG_, compared with PD patients without FOG (PD_NOFOG_) in simple gait condition (Snijders et al., 2011). With similar techniques, Peterson et al. (2014) found that neural activities in the supplementary motor area (SMA) and striatum were increased in PD_NOFOG_ during MI of simple walking (Peterson et al., 2014). The aforementioned illustrated the feasibility of adopting fMRI and MI for the investigation of FOG in PD. These studies are intriguing, but two issues remain to be elucidated. First, no normal controls were used for comparison with the patients, and it remains uncertain whether the results were disease-specific. Second, the MI paradigms adopted only pure imagery with no explicit guidance. As a result, it was difficult to verify how the participants could be imagining the gait conditions with and without freezing during the experimental process.

In this current study, we adopted a novel fMRI design of MI to study FOG using the first-person perspective video clips (Iseki et al., 2008; Wang et al., 2009) of multiple gait conditions, including forward straight walking and 360° turning, with and without FOG. We aimed to investigate (A) the cerebral blood oxygen level-dependent (BOLD) responses to different gait conditions among PD_FOG_, PD_NOFOG_, and normal controls, and (B) the patterns of cerebral BOLD activation between simple (forward straight) and complex (turning) walking. We combined the activation analysis and ROI analyses of the three locomotion regions [MLR, cerebellar locomotion region (CLR), and subthalamic locomotion region (SLR)] to analyze the fMRI data to gain sensitivity and preserve the statistical power of data analysis.

## Materials and Methods

### Participants

Thirty-seven PD patients aged between 50 and 80 years were enrolled in this study (58.8% male; mean age 67.6 ± 7.1 years). All participants fulfilled the UK Brain Bank Criteria for idiopathic PD (Gibb et al., 1990). Among the participants, 22 PD patients who obtained 1 point from Part I of the New FOG Questionnaire (nFOG-Q) (Nieuwboer et al., 2009) and experienced at least one FOG episode during gait assessment (see clinical and gait assessment) were labeled PD_FOG_, whereas the other 15 PD patients who had never experienced a FOG attack were grouped as PD_NOFOG_. Fifteen age- and education-level-matched normal controls were also recruited (33.3% male; mean age 63.4 ± 7.0 years; see Table 1). To confirm the MI ability during fMRI of each participant, the Vividness of Motor Imagery Questionnaire (VMIQ) (Isaac et al., 1986) was administered before recruitment. According to the previous report (Snijders et al., 2011), poor MI ability was defined as VMIQ scores >200, and no participants were excluded from this study due to VMIQ scores >200. Each PD patient was then investigated during the “off” state by withholding his/her medications overnight for over 12 h. Informed consent was obtained before the investigation. The study was approved by the Local Ethics Committee of the hospital in accordance with the Declaration of Helsinki (CMUH106-REC2-171) and was registered on ClinicalTrials.gov, with the identifier number NCT03127475.

**Table 1:** Demographic characteristics of PD_FOG_, PD_NOFOG_, and normal controls.

	**PD_FOG_ (*N* = 20)** **Mean ± SD**	**PD_NOFOG_ (*N* = 14)** **Mean ± SD**	**Normal control (*N* = 15)** **Mean ± SD**	***p*** **-value^†^**	***p*** **-value^‡^**
Age	66.0 ± 6.2	69.8 ± 7.8	63.4 ± 7.0	0.055	0.126
Gender (% male)	60%	57.1%	33.3%	0.177	1.000
Education (years)	9.8 ± 3.5	8.6 ± 3.9	9.2 ± 3.7	0.666	0.376
MMSE	28.3 ± 2.0	27.1 ± 2.5	28.9 ± 1.1	0.050	0.126
VMIQ score	64.8 ± 31.2	57.2 ± 38.6	56.1 ± 26.7	0.574	0.696
UPDRS	51.3 ± 20.1	37.9 ± 18.0			0.056
UPDRS-III	30.4 ± 15.2	24.4 ± 14.1			0.254
H&Y	3.1 ± 0.7	2.2 ± 0.5			<0.001^***^
LEDD (mg/day)	775.6 ± 404.3	580.6 ± 303.4			0.137
Disease duration (years)	8.1 ± 4.8	5.9 ± 2.7			0.143
PDQ-39	38.1 ± 16.7	24.4 ± 19.1			0.033^*^
nFOG-Q	20.7 ± 5.8	0			NA
Velocity (cm/s)	41.6 ± 25.5^•^^◦^	68.9 ± 22.4^⊚^^◦^	102.6 ± 16.3^⊚^^•^	< 0.001^***^	
Cadence (steps/min)	96.48 ± 24.9	103.9 ± 19.0	107.9 ± 8.5	0.181	
Stride length	50.5 ± 26.9^•^^◦^	78.6 ± 19.3^⊚^^◦^	113.3 ± 12.0^⊚^^•^	< 0.001^***^	
Stride width	13.7 ± 3.8	13.9 ± 5.4	12.4 ± 3.2	0.563	
Gait cycle	1.4 ± 0.5	1.2 ± 0.3	1.1 ± 0.1	0.084	
Stance time (%)	73.7 ± 6.9^•^^◦^	66.2 ± 3.4◦	63.6 ± 1.6^•^	< 0.001^***^	
Swing time (%)	26.3 ± 6.9^•^^◦^	33.8 ± 3.4^◦^	36.4 ± 1.6^•^	< 0.001^***^	
Single support time (%)	27.6 ± 5.8^•^^◦^	33.6 ± 3.1^◦^	36.1 ± 1.5^•^	< 0.001^***^	
Total double support time (%)	49.1 ± 14.3^•^^◦^	32.5 ± 6.3^◦^	27.1 ± 3.0^•^	< 0.001^***^	

† *Comparison among PD_FOG_, PD_NOFOG_, and normal controls.*

‡ *Comparison between PD_FOG_ and PD_NOFOG_.* *PD_FOG_, Parkinson's disease with FOG; PD_NOFOG_, Parkinson's disease without FOG; SD, standard deviation; MMSE, Mini-Mental State Examination; VMIQ, the Vividness of Motor Imagery Questionnaire; UPDRS, the Unified Parkinson's Disease Rating Scale; UPDRS-III, the Unified Parkinson's Disease Rating Scale motor subsection; H&Y, Hoehn–Yahr staging; LEDD, levodopa equivalent daily dose; R, right; L, left; PDQ-39, the Parkinson's Disease Questionnaire; nFOG-Q, New Freezing of Gait Questionnaire.*

◦ *Significant difference between PD_FOG_ and PD_NOFOG_ (p < 0.016).*

• *Significant difference between PD_FOG_ and normal controls (p < 0.016).*

⊚ *Significant difference between PD_NOFOG_ and normal controls (p < 0.016).*

* *p < 0.05 and*

*** *p < 0.001*.

### Clinical and Gait Assessment

The clinical assessments used are as follows: the Unified Parkinson's Disease Rating Scale (UPDRS) (Gibb and Lees, 1988), the UPDRS motor subsection (UPDRS-III), Hoehn–Yahr (H&Y) staging, levodopa equivalent daily dose (LEDD) (Tomlinson et al., 2010), Parkinson's Disease Questionnaire (PDQ-39) (Rahman et al., 2008b), nFOG-Q (Nieuwboer et al., 2009), and the Mini-Mental State Examination (MMSE) (Folstein et al., 1975).

For the gait assessment, the participants were instructed to walk back and forth on a 5-m walkway for five laps (50 m in total) in the gait analysis laboratory equipped with a Zeno 16 × 2 feet pressure-sensitive carpet. Concomitant video clips were also recorded throughout the entire gait assessment. Freezing episodes were identified by two independent, experienced neurologists from the video clips. The gait parameters were then analyzed by Protokinetics Movement Analysis System 5.08 (Peekskill, NY, United States). These parameters included velocity (cm/s), cadence (steps/min), stride length (cm) (Chaudhuri et al., 2007), stride width (cm) (Chaudhuri et al., 2007), gait cycle (Cugusi et al., 2017), stance time (% of gait cycle), swing time (% of gait cycle), single support time (% of gait cycle), and total double support time (% of gait cycle). We also analyzed the correlation between the nFOG-Q score of PD_FOG_ and gait parameters using Spearman's rank-order correlation coefficient test. The threshold for statistical significance was set at bivariate *p* < 0.05.

### Functional Magnetic Resonance Imaging Experiment

#### Paradigm of MI

**Figure 1:**
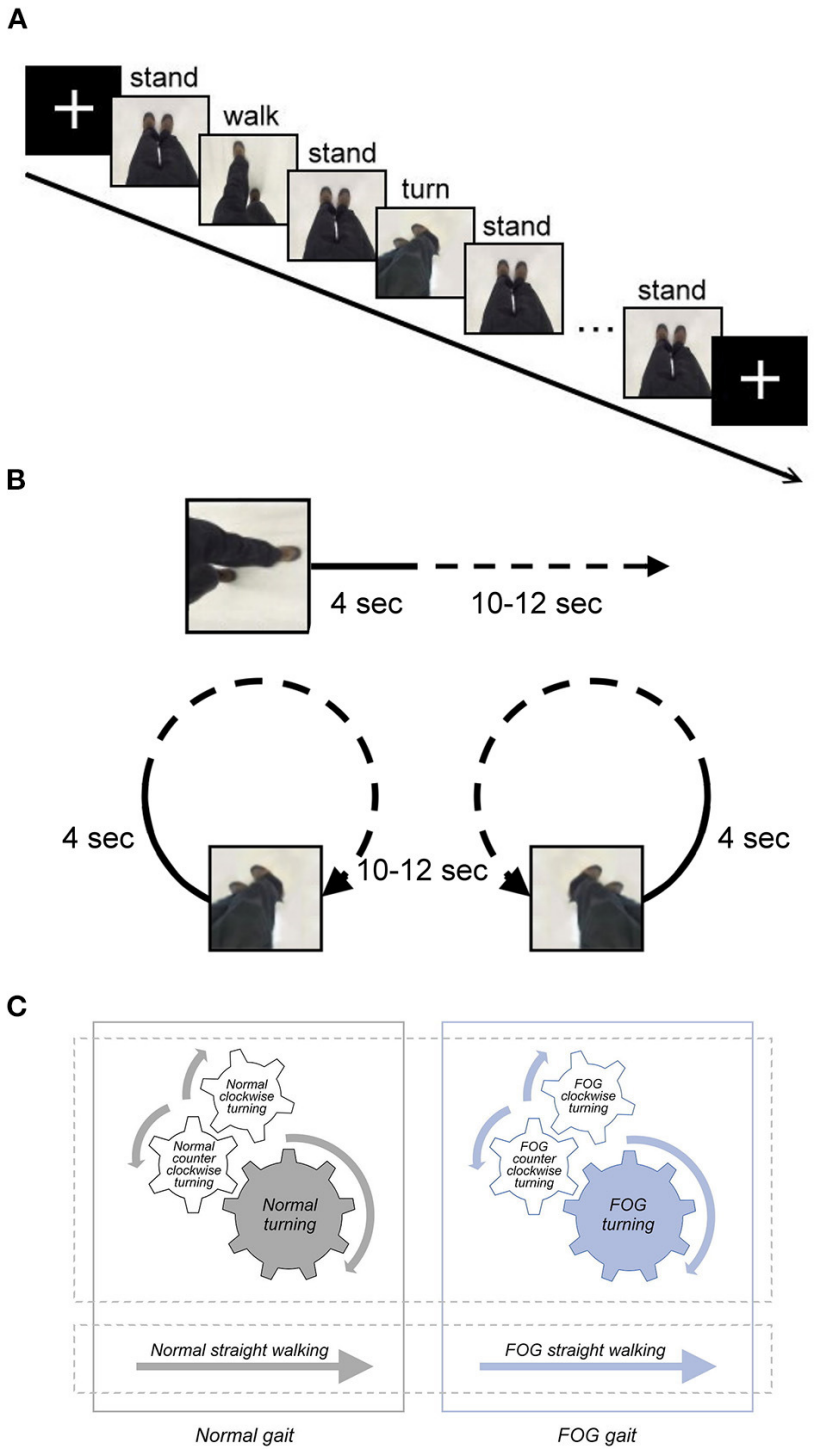
**(A)** Graphical representation of the fMRI paradigm, including snapshots from the gait video clips to represent the stimulus. The duration of the stimuli, including *normal straight walking, FOG straight walking* (not shown in this figure), *normal clockwise turning* (not shown), *normal counter-clockwise turning, FOG clockwise turning* (not shown), and *FOG counter-clockwise turning* (not shown), varied between 14 and 16 s. The duration of the fixed cross and standing stimuli was 5 s. **(B)** Graphical representation of the video of FOG condition. The video clip started with normal walking or turning for 4 sec, followed by 10–12 s of FOG walking or turning (70 % freezing in each FOG condition). **(C)** Diagram illustrating the content relationship among various gait conditions in the study. *Normal clockwise turning* combined with *normal counter-clockwise turning* together as *normal turning*; *FOG clockwise turning* combined with *FOG counter-clockwise turning* as *FOG turning*. All FOG-related conditions, including *FOG straight walking* and *FOG turning*, were jointly referred to as the *FOG gait* condition, and all the normal conditions were jointly referred to as *normal gait*.

For the MI fMRI experiment, two fMRI sessions were conducted with a blocked experimental design. Each session comprised six pseudorandomized blocks of video clips of different gait conditions, including three types of walking (straight walking, 360° clockwise turning, and 360° counter-clockwise turning) with and without FOG (Figure 1). As a result, each participant watched the video clips of six gait stimulus conditions (*normal straight walking, normal clockwise turning, normal counter-clockwise turning, FOG straight walking, FOG clockwise turning*, and *FOG counter-clockwise turning*). The video clips recorded the same actor, viewing from the top and watching his ambulating feet in a first-person perspective, walking in six conditions. The velocities of *normal straight walking* and *normal turning* were 100 cm/s and 25°/s, respectively. The FOG condition was a video clip starting with normal walking or turning for 4 s, followed by FOG walking or turning (70% freezing in each FOG condition; Figure 1B). Each gait stimulus video clip lasted for 14–16 s. In between the consecutive gait blocks, a 5-s “standing” video clip was used as a baseline condition for further contrasting different gait conditions. The total duration of each functional session was 425 s, with counterbalanced order for the six gait conditions across participants.

#### Image Acquisition

The fMRI/MRI data were collected using a 3.0 T MR system (Signa HDxt, GE, Milwaukee, WI, United States). The video clips of different gait conditions were projected on the screen installed at the rear side of the MR scanner. Each participant watched the video through the reflective mirror mounted on the head coil and mentally imagined themselves performing the gait action currently played during the functional experiment. T2^*^-weighted echo planar functional images were acquired in an interleaved slice order. Other imaging parameters included were as follows: TR = 2 s, TE = 35 ms, flip angle = 90°, 32 axial slices covering the whole brain, field of view = 224 × 224 mm^2^, matrix = 64 × 64, and slice thickness = 4 mm with 0.4 mm inter-slice gap. This resulted in an effective image resolution of 3.5 × 3.5 × 4.4 mm in voxel size. High-resolution 3D T1-weighted anatomical images (voxel size 1 × 1 × 1 mm) were obtained using a three-dimensional inversion recovery prepped spoiled gradient recalled echo pulse sequence for co-registering the functional data to the individual structure and finally the standard brain template.

#### Data Analysis of fMRI

Pre-processing and activation analysis of the fMRI data were performed using Statistical Parametric Mapping 8 (SPM8) software (the Wellcome Trust Centre for Neuroimaging, London, United Kingdom) running under MATLAB R2014b (MathWorks, Inc., Boston, MA United States). The pre-processing steps included slice timing correction, realignment, co-registration, spatial normalization, and spatial smoothing. Among these pre-processing steps, Montreal Neurological Institute coordinate space was used in the spatial normalization, and a Gaussian filter with an 8-mm full-width at half-maximum was used in the spatial smoothing (Evans et al., 1991, http://www.bic.mni.mcgill.ca).

##### General Linear Model

In this study, we used general linear model (GLM) (Friston et al., 1994), which includes linear multiple regression, to find the voxels with their BOLD time courses highly in line with the task-related reference function, with other nuisance factor, such as the six degree-of-freedom motion parameters, etc., controlled in the GLM. This is so-called the activation analysis at the single-subject level for the active cerebral regions under the contrast of different gait conditions. Then, directional *t*-test (also called two-sample *t*-test) for comparing each set of two groups (PD_FOG_ vs. controls, PD_NOFOG_ vs. controls, and PD_FOG_ vs. PD_NOFOG)_ were performed during group analysis. This statistical comparison procedure also highly complied with what had been reported in the literature (Poline and Brett, 2012). The GLM contains forms of experimental layout and ensuing statistical analysis including linear regression and ANOVA. This approach connects the general linear model and the theory of Gaussian fields to give a simple and complete framework for the analysis of imaging data. In this study, GLMs (Friston et al., 1994, 1995) were estimated with a high-pass filter of 128 s.

### Statistical Analysis

#### Activation Analysis at the Single-Subject Level

The pre-processed fMRI time-series data were analyzed on a subject-by-subject basis using a blocked design approach in the context of the GLM to find the cerebral regions responding to the contrasts of different gait conditions. The contrasts of *normal straight walking* > standing (baseline), *FOG straight walking* > standing, *normal clockwise turning* > standing, *normal counter-clockwise turning* > standing, *FOG clockwise turning* > standing, and *FOG counter-clockwise turning* > standing were used in the statistical analysis. Directional T-contrasts for comparison of each group were estimated. The threshold value for statistical significance was set to *p* < 0.05 after multiple-comparison correction by false discovery rate (FDR) at the voxel level (Genovese et al., 2002).

**Figure 2:**
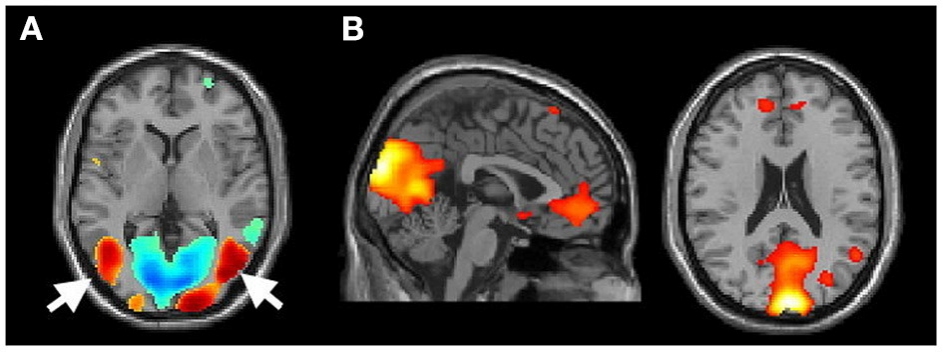
**(A)** Activation of the MT/V5 area (arrow) of participant 11 (P11) while performing the motor imagery task during fMRI and **(B)** activation of the default mode (from the excluded P3).

To ensure that the participants were fully engaged in the MI task, we have carefully reviewed the activation of the GLM analysis at the single-subject level. Further, those participants who showed no activation in the motion-sensitive visual areas (e.g., the MT/V5 areas, Figure 2A) (Kaas et al., 2010; Kolster et al., 2010), but pronounced default mode network activation (Figure 2B), indicating that they might not actively watch the video clips in the scanner, were further excluded from the latter group analysis. Such a criterion excluded two PD_FOG_ patients and one PD_NOFOG_ patient from further analysis.

#### Group Analysis

Given that the activation analysis at the single-subject level did not differ between clockwise and counter-clockwise turning, we opted to combine the conditions of clockwise and counter-clockwise turning to form the conditions of *normal turning* and *FOG turning* for the group analysis to increase the data trial number and improve the statistical power. Further, all FOG-related conditions, such as the *FOG straight walking* and *FOG turning*, were combined into the *FOG gait* condition, and all normal conditions were also combined as *normal gait* (Figure 1C).

For each participant, six contrasts (*normal straight walking, FOG straight walking, normal turning, FOG turning, normal gait*, and *FOG gait*) were conducted. Furthermore, to examine the different cerebral activation patterns between simple gait and complex gait, additional two contrasts, namely, *normal turning* > *normal straight walking* and *FOG turning* > *FOG straight walking*, were also conducted. Directional T-contrasts for comparing each set of two groups (PD_FOG_ vs. controls, PD_NOFOG_ vs. controls, and PD_FOG_ vs. PD_NOFOG_) were performed. Given that the H&Y staging was higher in PD_FOG_ than in PD_NOFOG_, the resulting contrasts from the two PD groups (PD_FOG_ vs. PD_NOFOG_) were further statistically compared against the H&Y staging as a covariate during group analysis to minimize the interference of the fMRI result by PD severity. The threshold value for statistical significance was set to *p* < 0.05 after correction by FDR at the voxel level. Furthermore, as the differences between each set of two groups when compared contrasts with *normal turning* > *normal straight walking* and *FOG turning* > *FOG straight walking* were too small when statistical significance was set to *p* < 0.05 after multiple-comparison correction by FDR at the voxel level, a statistically significant threshold of *p* < 0.001 (without multiple-comparison correction) was used when comparing these two contrasts.

#### Analysis of ROI

In addition to the activation analysis mentioned above, we have further compared the BOLD activation of the three locomotion regions, namely, the MLR, CLR, and SLR, to investigate the role of these three areas in PD_FOG_, PD_NOFOG_, and normal controls in different gait conditions. Due to their small sizes, these three brain areas failed to show a significant activation in the regular activation analysis. As a result, we delineated these three brain areas based on the coordinates as reported in the previous literature, that is (x = ±7, y = −52, z = −16), for the CLR; (x = ±4, y = −30, z = −16), for the MLR; and (x = ±11, y = −14, z = −3) for the SLR, bilaterally (Snijders et al., 2011; Fling et al., 2014). A 4-mm radius for the MLR and an 8-mm radius for the CLR and the SLR surrounding the designated coordinates were used to extract the effect sizes (beta values) of the three ROIs using the MarsBaR toolbox in SPM8.

The mean effect sizes (beta values) were then extracted and averaged from the designated ROIs and then imported into SPSS v.19 (SPSS, Inc., Chicago, IL, United States) for the group-level analysis. One-way ANOVA was performed to determine whether each of the ROIs was significantly associated with the contrast of *normal straight walking, FOG straight walking, normal turning, FOG turning, normal gait*, and *FOG gait*. Furthermore, the effect size from each ROI of the PD_FOG_ was compared against the nFOG-Q score, whereas the effect size from each ROI of the PD population (PD_FOG_ and PD_NOFOG_, respectively), was compared against the H&Y staging, PD disease duration, and gait parameters from the gait assessment using bivariate Spearman's rank-order correlations. The threshold for statistical significance was set at *p* < 0.05.

## Results

### Results of Clinical and Gait Assessments

Table 1 shows the demographic characteristics of the participants. There were no significant differences in age, gender, number of years of education, VMIQ, and MMSE among PD_FOG_, PD_NOFOG_, and normal controls. There were also no significant differences for UPDRS, UPDRS-III, LEDD, and disease duration between PD_FOG_ and PD_NOFOG_. Moreover, no significant differences were noted in the motor symptom lateralization in PD_FOG_ (seven left-sided and seven right-sided predominating) and PD_NOFOG_ (13 left-sided and seven right-sided predominating, with no significant difference in the summation of UPDRS motor score on each side; left-sided, 6.9 ± 4.8; right-sided, 6.3 ± 4.6; *p* = 0.991). However, PD_FOG_ showed more severe H&Y staging and PDQ-39 than PD_NOFOG_.

Significant differences were observed in the velocity, stride length, stance time, swing time, single support time, and total double support time among PD_FOG_, PD_NOFOG_, and normal controls (Table 1). PD_FOG_ had the slowest gait, smallest stride length, increased stance time, decreased swing time, least single support time, and increased total double support time. Furthermore, the nFOG-Q score in PD_FOG_ was significantly positively correlated with stance time (ρ = 0.492, *p* = 0.032) and total double support time (ρ = 0.506, *p* = 0.023), whereas it was significantly negatively correlated with velocity (ρ = −0.505, *p* = 0.023), swing time (ρ = −0.492, *p* = 0.032), and single support time (ρ = −0.45, *p* = 0.047). No significant correlations were found between the nFOG-Q score and cadence (ρ = −0.358, *p* = 0.121), stride length (ρ = −0.374, *p* = 0.105), stride width (ρ = 0.069, *p* = 0.774), and gait cycle (ρ = 0.345, *p* = 0.136).

### Imaging Results

#### Activation Analysis

##### Different Patterns of Cerebral Responses of Normal Gait and FOG Gait Conditions Among PD_**FOG**_, PD_**NOFOG**_, and Normal Controls

**Figure 3:**
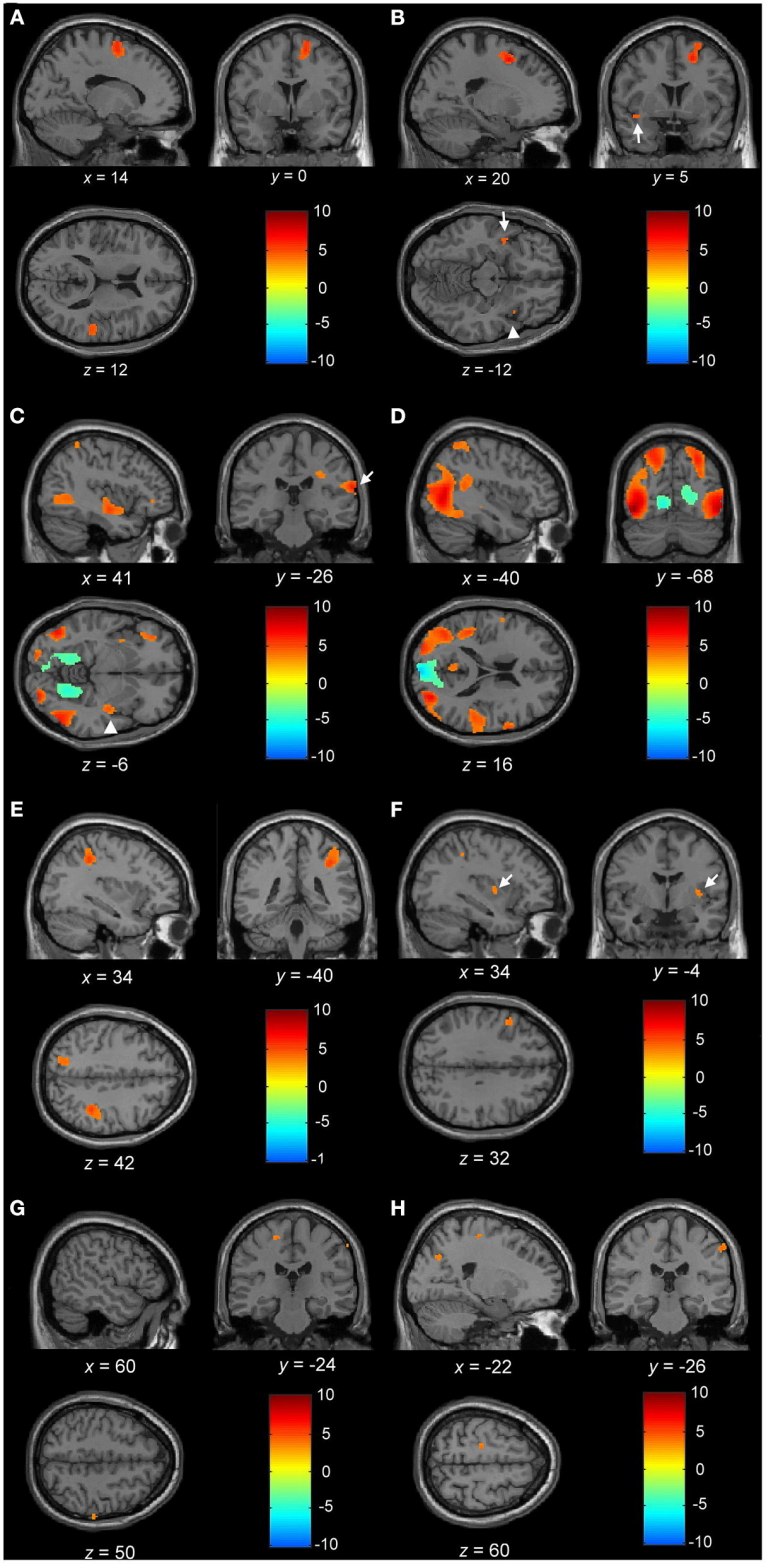
**(A)** Blood oxygen level-dependent (BOLD) activation during motor imagery (MI) of *normal gait* with the contrast of normal controls to PD_NOFOG_. Increased BOLD in the right SMA, right superior temporal gyrus, left SMA (not shown), and right medial superior frontal gyrus (not shown). **(B)** BOLD activation during MI of *FOG gait* with the contrast of PD_FOG_ to PD_NOFOG_. Increased BOLD in the right superior and middle frontal gyrus, right insula (arrowhead), left superior temporal cortex (arrow), and left superior frontal gyrus (not shown). **(C)** BOLD activation during MI of *FOG straight walking* in PD_FOG_. Increased BOLD in the right insula (arrowhead), right postcentral gyrus (arrow), right middle temporal, left middle occipital, and right inferior occipital lobule. **(D)** BOLD activation during MI of *FOG turning* in PD_FOG_. Increased BOLD in the right precentral gyrus, right middle temporal, left superior temporal, bilateral superior parietal, left middle and inferior occipital, and right superior occipital lobule. **(E)** BOLD activation during MI of *FOG turning* > *FOG straight walking* in PD_FOG_. Increased BOLD in the right superior and inferior parietal, left superior parietal lobule, and right middle occipital lobule (not shown). **(F)** BOLD activation during MI of *normal turning* > *normal straight walking* in PD_FOG_ > controls. Increased BOLD in the left inferior frontal, right putamen (arrow), and right inferior parietal lobule. **(G)** BOLD activation during MI of *FOG turning* > *FOG straight walking* in control > PD_NOFOG_. Increased BOLD in the left precentral and right postcentral gyrus. **(H)** BOLD activation during MI of *FOG turning* > *FOG straight walking* in PD_FOG_ > PD_NOFOG_. Increased BOLD in the left precentral, right postcentral gyrus, and left superior occipital lobule. **(A–E)** Statistical maps were created and corrected for the false discovery rate (FDR) at *p* < 0.05 at the voxel level. **(F–H)** Statistical maps were created at *p* < 0.001 (without multiple-comparison correction).

*Normal Gait*. The results comparing the BOLD responses of *normal gait* among PD_FOG_, PD_NOFOG_, and normal controls have revealed significantly higher activation in the bilateral SMA, right superior temporal, and right medial superior frontal gyrus in normal controls than PD_NOFOG_ (Table 2 and Figure 3A). Moreover, a significant activation was observed in the right SMA in normal controls compared with PD_NOFOG_ during the *normal straight walking* (Table 2). No significant difference was detected in the brain activations associated with *normal turning* among the three groups.

**Table 2:** Results from the blocked design analysis.

**Task**	**Contrast**	**Localization**	**Hemisphere**	**x**	**y**	**z**	***p*** **-value^†^**
Normal gait	Control > PD_NOFOG_	SMA	Right	14	0	66	0.014^*^
		Superior temporal	Right	52	−30	14	0.028^*^
		SMA	Left	−2	−10	62	0.036^*^
		Medial superior frontal	Right	6	34	56	0.041^*^
		Superior temporal pole	Right	50	16	−20	0.049^*^
Normal straight walking	Control > PD_NOFOG_	SMA	Right	10	0	52	0.044^*^
FOG gait	PD_FOG_ > PD_NOFOG_	Superior frontal	Right	18	8	52	0.009^**^
		Insula	Right	36	12	−12	0.049^*^
		Superior temporal	Left	−44	4	−12	0.045^*^
		Middle frontal	Right	44	12	50	0.045^*^
		Superior frontal	Left	−20	−2	54	0.049^*^
FOG straight walking	PD_FOG_	Inferior occipital	Right	26	−92	−2	< 0.001^***^
		Middle occipital	Left	−48	−72	−2	< 0.001^***^
		Middle occipital	Left	−18	−98	0	0.001^**^
		Middle temporal	Right	52	−70	−2	< 0.001^***^
		Post-central gyrus	Right	64	−26	20	< 0.001^***^
		Insula	Right	40	−10	−10	0.002^**^
FOG turning	PD_FOG_	Precentral gyrus	Right	62	8	24	< 0.001^***^
		Superior temporal	Left	−44	−42	14	< 0.001^***^
		Middle temporal	Right	48	−68	−2	< 0.001^***^
		Superior parietal	Right	26	−76	54	< 0.001^***^
		Superior parietal	Left	−24	−66	66	< 0.001^***^
		Superior occipital	Right	28	−80	18	< 0.001^***^
		Middle occipital	Left	−30	−90	18	< 0.001^***^
		Inferior occipital	Left	−48	−70	−4	< 0.001^***^
FOG turning >	PD_FOG_	Inferior parietal	Right	34	−40	42	0.02^*^
FOG straight walking		Superior parietal	Right	22	−60	60	0.02^*^
		Superior parietal	Left	−16	−72	54	0.02^*^
		Middle occipital	Right	30	−78	22	0.049^*^
	PD_FOG_ > PD_NOFOG_	Postcentral	Right	58	−26	48	<0.001^***^^#^
		Precentral	Left	−20	−22	60	<0.001^***^^#^
		Superior occipital	Left	−22	−70	34	<0.001^***^^#^
	Control > PD_NOFOG_	Precentral	Left	−20	−22	60	<0.001^***^^#^
		Postcentral	Right	60	−26	50	<0.001^***^^#^
Normal turning >	PD_FOG_ > Control	Inferior frontal	Left	−52	14	32	<0.001^***^^#^
Normal straight walking		Putamen	Right	32	−4	6	<0.001^***^^#^
		Inferior parietal	Right	36	−40	46	<0.001^***^^#^

† *Corrected for the false discovery rate (FDR) at the voxel level.*

# *p < 0.001 (without multiple-comparison correction) at the voxel level.*

* *p < 0.05,*

** *p < 0.01, and*

*** *p <0.001*.

*FOG Gait*. During the *FOG gait*, a significant activation occurred in the bilateral superior frontal, right middle frontal, right insula, and left superior temporal gyrus of PD_FOG_ compared with PD_NOFOG_ (Table 2 and Figure 3B). No significant difference was found in the brain activation associated with *FOG turning* and *FOG straight walking* among the three groups.

By only considering the PD_FOG_ patient data, the increased cerebral activation of the right insula, right postcentral gyrus, right middle temporal, left middle occipital, and right inferior occipital lobule was detected in *FOG* straight *walking* (Table 2 and Figure 3C). The increased cerebral activation of the right precentral gyrus, right middle temporal, left superior temporal, bilateral superior parietal, left middle and inferior occipital, and right superior occipital was detected in *FOG turning* (Table 2 and Figure 3D). By subtracting *FOG straight walking* cerebral activation from that of *FOG turning*, the right superior and inferior parietal, left superior parietal lobule, and right middle occipital activation were retained (Table 2 and Figure 3E).

#### Analysis of ROI of the Locomotion Regions

##### Different Patterns of Locomotion Region Responses Between Normal Gait and FOG Gait Conditions Among PD_**FOG**_, PD_**NOFOG**_, and Normal Controls

**Figure 4:**
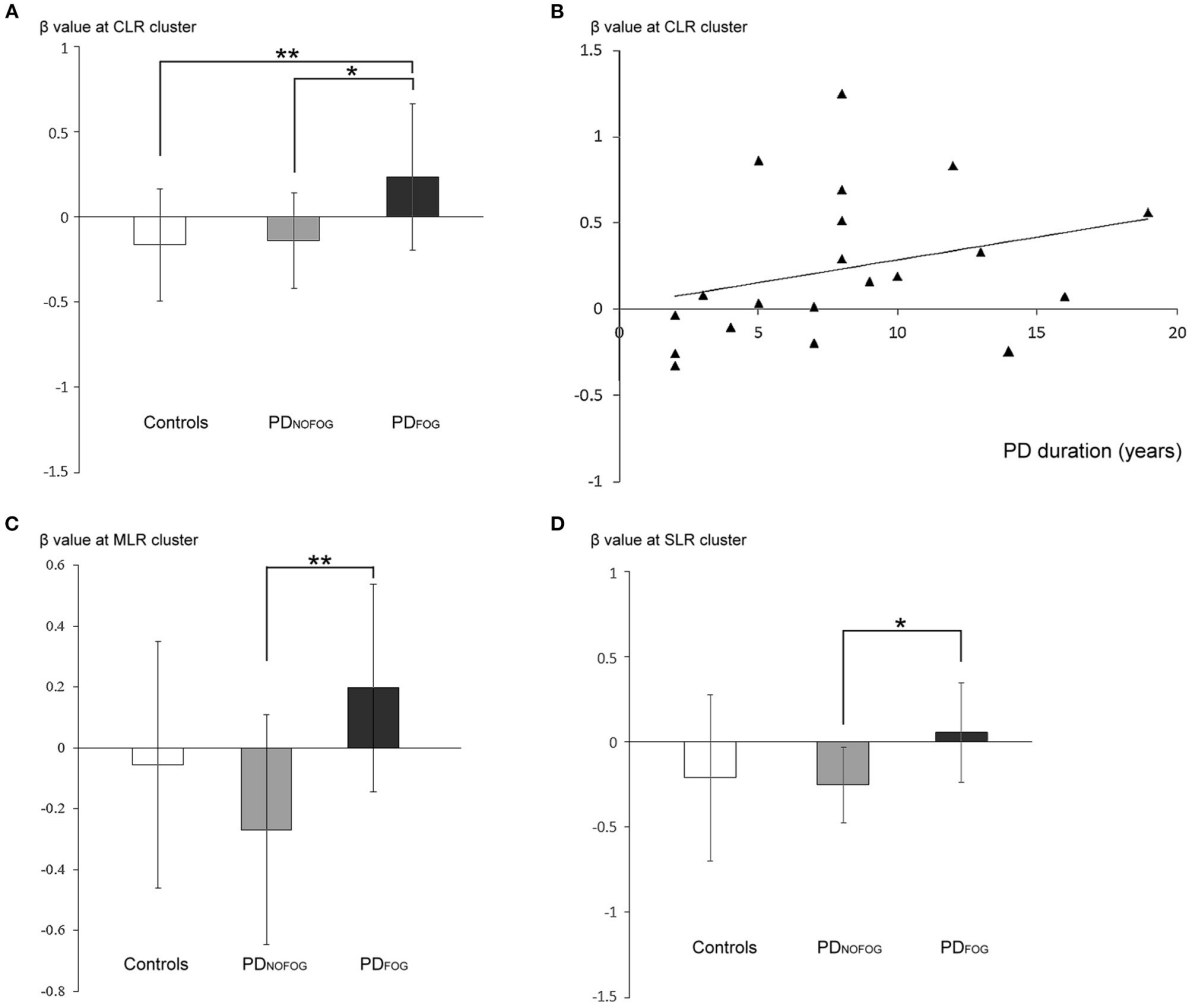
**(A)** β-weights of the contrast between *normal turning* and standing (mean ± standard deviation) from the cerebellar locomotor region (CLR) clusters in normal controls, PD_NOFOG_, and PD_FOG_. **(B)** Scatterplots of β-weights at the CLR cluster (y-axis) against PD duration (x-axis; Spearman's correlation ρ = 0.455, *R*^2^ = 0.088, *p* = 0.044) during *normal turning* in PD_FOG_. **(C)** β-weights of the contrast between *normal turning* and standing (mean ± standard deviation) from the mesencephalic locomotor region (MLR) clusters in normal controls, PD_NOFOG_, and PD_FOG_. **(D)** β-weights of the contrast between *normal turning* and standing (mean ± standard deviation) from the subthalamic locomotor region (SLR) clusters in normal controls, PD_NOFOG_, and PD_FOG_. *p < 0.05, **p < 0.01.

*Increased Beta Value of Locomotion Regions During *Normal Turning* but Not *FOG turning* in PD_FOG_.* Analysis of ROI of the CLR activity against different gait conditions among PD_FOG_, PD_NOFOG_, and normal controls revealed a significantly higher beta value of CLR in PD_FOG_ than in controls and PD_NOFOG_ during the *normal turning* conditions (*p* = 0.003; PD_FOG_ vs. controls, *p* = 0.007; PD_FOG_ vs. with PD_NOFOG_, *p* = 0.015; all FDR-corrected, Figure 4A). No significant difference was noted during *FOG turning* (*p* = 0.394).

The ROI analysis of the MLR activity against different gait conditions among PD_FOG_, PD_NOFOG_, and normal controls revealed a significantly higher beta value of MLR in PD_FOG_ compared with PD_NOFOG_ during the *normal turning* conditions (*p* = 0.003, FDR-corrected, Figure 4C). No significant difference was detected during *FOG turning* (*p* = 0.14).

The ROI analysis of the SLR activity against different gait conditions among PD_FOG_, PD_NOFOG_, and normal controls revealed a significantly higher beta value of SLR in PD_FOG_ than in PD_NOFOG_ during the *normal turning* conditions (*p* = 0.023, FDR-corrected, Figure 4D). No significant difference was detected during *FOG turning* (*p* = 0.084).

#### Complex (Turning) vs. Simple Gait (Forward Straight Walking) in Normal Gait and FOG Gait Among PD_FOG_, PD_NOFOG_, and Normal Controls

##### Results of the Activation Analysis

When comparing complex to simple gait *via* conducting contrast of *normal turning* > *normal straight walking* and *FOG turning* > *FOG straight walking* among PD_FOG_, PD_NOFOG_, and normal controls, no significant difference was noted between each group using the statistical significance threshold of *p* < 0.05 (FDR-corrected). Therefore, we lowered the statistical significance to *p* < 0.001 (without multiple-comparison correction) and showed the subtle differences among the three groups. While comparing *normal turning* > *normal straight walking* among groups, PD_FOG_ showed an increased activation of the left inferior frontal, right putamen, and right inferior parietal lobule when compared with controls (Table 2 and Figure 3F).

Furthermore, while comparing *FOG turning* > *FOG straight walking* among the three groups, controls showed an increased activation of left precentral and right postcentral gyrus compared with PD_NOFOG_ (Table 2 and Figure 3G). In addition, PD_FOG_ showed an increased activation of the left superior occipital gyrus, left precentral gyrus, and right postcentral gyrus when compared with PD_NOFOG_ (Table 2 and Figure 3H).

##### Results of ROI

No significant difference in the beta values from ROI analysis of the MLR, CLR, or SLR activity of *normal turning* > *normal straight walking* and *FOG turning* > *FOG straight walking* was observed within each individual group (*normal turning* > *normal straight walking*: CLR, *p* = 0.07; MLR, *p* = 0.16; SLR, *p* = 0.92. *FOG turning* > *FOG straight walking*: CLR, *p* = 0.26; MLR, *p* = 0.11; SLR, *p* = 0.74).

#### Relationships Between the Beta Values of Locomotion Regions and Disease Severity

The beta values of CLR activity during *normal turning* in PD_FOG_ were significantly positively correlated with PD disease duration (Figure 4B; *p* = 0.044). Moreover, no correlation was found between the beta values of the CLR activity and disease severity (UPDRS, *p* = 0.705; UPDRS-III, *p* = 0.84; H&Y, *p* = 0.647; and nFOG-Q, *p* = 0.845). Furthermore, no correlation was found between the beta values of the MLR or SLR activity and disease severity, and no significant correlation was detected between the beta values of the CLR, MLR, or SLR and gait parameters either.

## Discussion

This study used MI of different gait conditions to investigate neural substrates of FOG. The “first-person-perspective” video, including normal/FOG straight walking and normal/FOG turning, was used to explicitly guide the MI. In addition, we have also measured the basic gait performance and clinical features of all subjects. We found that during imagery of *normal gait*, the PD_NOFOG_ had a significantly reduced BOLD response in the bilateral SMA, right superior temporal, and right medial superior frontal gyrus relative to the controls. During MI of *FOG gait*, the greater BOLD response was found in the bilateral frontal lobe, left superior temporal lobe, and right insula of the PD_FOG_ than in those of the PD_NOFOG_. Furthermore, PD_FOG_ manifested a higher beta value in the CLR than in controls and PD_NOFOG_ and a higher beta value in the MLR and SLR than in PD_NOFOG_ during imagery of *normal turning*. The cerebral activity during complex gait was more activated than simple gait in PD_FOG_.

### Less Gait-Related Cerebral Activation in PD_NOFOG_ Than in PD_FOG_ and Normal Controls

Overt cerebral deactivation during the MI of different gait conditions was observed in PD_NOFOG_. In particular, the activity of bilateral SMA and right frontal and right superior temporal lobules in PD_NOFOG_ decreased compared with normal controls during *normal gait* MI. These findings are consistent with the results of an action observation fMRI study of normal walking in PD (Bommarito et al., 2020). From the physiological point of view, the SMA and premotor cortex have a tight connection to the spinal cord and brainstem reticular formation, including the pedunculopontine nucleus (PPN) area and MLR (Aravamuthan et al., 2009). Thus, the SMA has been identified to be crucial for gait initiation and postural control (Jacobs et al., 2009). Moreover, changes in body schema information originating from the temporoparietal cortex to the frontal cortex might promote motor programming in the SMA and premotor cortex to improve postural control and anticipatory postural adjustment during bipedal locomotion (Takakusaki, 2013). Furthermore, the basal ganglia control the locomotion and posture through the GABAergic pathway from the substantia nigra reticulata that blocks the PPN-induced muscle atonia and the MLR-induced locomotion (Takakusaki et al., 2003). However, the overinhibition from the basal ganglia in PD patients might cause them to experience gait disturbances, accounting for the perturbation of SMA, and frontal and temporal activities in PD_NOFOG_ (Jenkins et al., 1992).

### Different Freezing-Related Cerebral Activities Between PD_FOG_ and PD_NOFOG_

During MI of the *FOG gait*, a significant activation occurred in the bilateral superior frontal, right middle frontal, right insula, and left superior temporal gyrus of PD_FOG_ compared with PD_NOFOG_ (Table 2 and Figure 3B). The increased activity in the frontal lobe and insula was in line with the results of the study carried out by Shine et al. (2013) and might be associated with the increased demand for cognitive gait control in PD_FOG_ to overcome freezing while autonomic gait control was being blocked. Frontal executive dysfunction has been associated with freezing in PD_FOG_ because FOG is more likely to occur during dual tasking (Spildooren et al., 2010) and PD_FOG_ patients show a poor performance in set-shifting tasks (Rahman et al., 2008a; Naismith et al., 2010; Mckay et al., 2018). A functional near-infrared spectroscopy (fNIRS)-based study confirmed the increased activation of the frontal lobe before and during anticipated turns in PD_FOG_ (Maidan et al., 2015). This result is similar to the increased freezing-related frontal activity observed in PD_FOG_. Furthermore, during the freezing attack in PD_FOG_, the increased information with body schema from the temporoparietal cortex to the frontal cortex might help PD_FOG_ compensate and maintain anticipatory postural adjustment and locomotion (Takakusaki et al., 2003).

### Motor Imagery of *Normal Turning* but Not *FOG Turning* Distinguishes PD_FOG_ From PD_NOFOG_ and Controls

Our ROI analysis revealed a significantly higher beta value in the CLR in PD_FOG_ compared with controls and PD_NOFOG_ during MI of *normal turning*. However, the difference between PD_FOG_ with controls and PD_NOFOG_ was quenched when the patients performed *FOG turning* MI. It is currently uncertain if the increased *normal turning*-related CLR is a compensatory adaptation for overcoming FOG or a pathological maladaptation leading to FOG. Since the phenomenon was not observed when doing *FOG turning* MI, a compensatory adaptation seems likely. A recent study illustrated that the CLR activities increased after receiving a course of “adapted resistance training with instability” in PD_FOG_ patients (Vieira-Yano et al., 2021). The increased CLR activities of fMRI were associated with the dual-task cost on stride length improvement of freezers (Vieira-Yano et al., 2021). The notion strengthened the viewpoint that an increased CLR activity is a compensatory adaptation rather than a pathological maladaptation. We also noticed that increased *normal turning*-related CLR activity in PD_FOG_ was significantly positively correlated with PD disease duration. The phenomenon implies that along with the progression of PD, stronger CLR compensation might be required to catch up the normality of walking. There was no significant correlation between CLR activity and disease severity in PD_FOG._ This might be due to the parameters (UPDRS, UPDRS-III, H&Y, and nFOG-Q) we used for disease severity assessment could not vividly reflect the degree of dynamic gait disorders in PD_FOG_. The cerebellum was anatomically and functionally connected with brainstem structures involved in gait and balance control (Youn et al., 2015). Several recent studies have provided evidence on the increased cerebellar functional connectivity with the posterior cortical areas in PD_FOG_ (Bharti et al., 2019; Jung et al., 2020). These findings further support our notion that CLR might play a compensatory role in PD_FOG_.

Furthermore, a similar phenomenon was also encountered in the MLR and the SLR, with higher beta value in the MLR and SLR found in PD_FOG_ than in PD_NOFOG_ during MI of *normal turning*. It is likely that PD_FOG_ patients may require a high impulse drive of locomotion regions to maintain their non-freezing ambulation (Gratsch et al., 2019). la Fougère et al. reported that the CLR, MLR, and SLR activity increases during MI of planning and modulation of locomotion (La Fougere et al., 2010), which could partially explain the increased burden of the locomotion regions to modify the complex gait during *normal turning* imagery in PD_FOG_. Increased functional connectivity between SMA with MLR and CLR in PD_FOG_ compared with PD_NOFOG_ was also reported (Fling et al., 2014). However, the increased activation in the CLR, MLR, and SLR in PD_FOG_ might not sustain adequately to stop the ongoing freezing all the time, and FOG might breach the compensation intermittently throughout the course of walking (Gratsch et al., 2019). Furthermore, Mori et al. demonstrated that stimulation of the CLR can independently induce locomotion in decerebrate cats (Mori et al., 1999; Rahimpour et al., 2021). However, although stimulation of the SLR or MLR also could evoke locomotion in decerebrate cats, the coordination of the limbs was greatly disrupted and extensor rigidity was elicited (Orlovskii, 1970; Rahimpour et al., 2021). These findings suggest that the CLR may play a pivotal role in coordinating SLR and MLR for generating and monitoring locomotion (Rahimpour et al., 2021). The current findings of no significant difference between the PD_FOG_ and normal subjects in MLR and SLR activities as what we saw in CLR also strengthen the notion.

### Unique Complex (*Turning*) vs. Simple (*Straight Walking*) Walking Patterns of PD_FOG_

Turning requires more complex neural control than straight walking. While comparing *normal turning* to *normal straight walking* among groups, PD_FOG_ showed an increased activation of the left inferior frontal lobe, right putamen, and right inferior parietal lobule compared to controls. The parietal and frontal regions were responsible for attention shifts and generating a high-level perception of motion (Culham et al., 1998), whereas the putamen regulates movement preparation and execution. As a result, the increased putamen activity together with the frontal and parietal lobule in PD_FOG_ during *normal turning* in this study might indicate the requirement of attentive motion control in PD_FOG_ during complex gait. This might also explain why dual tasks easily trigger FOG attacks (Spildooren et al., 2010).

While comparing *FOG turning* to *FOG straight walking* condition, we found increased bilateral parietal and right occipital cerebral activities in PD_FOG_. In addition, an increased activation of the superior occipital lobule, together with the precentral and postcentral gyrus, was detected when PD_FOG_ was compared to PD_NOFOG_. These findings are in line with those observed by Piramide et al. (2020), suggesting that the dorsal visual pathway of the parieto-occipital networks may enhance the spatiotemporal demands during locomotion. As a result, an increased dorsal visual pathway activation might also play a compensatory role in overcoming the fronto-striatal failure in PD during complex walking (Piramide et al., 2020). This condition might also explain why visual cue or action observation training could attenuate freezing attacks (Agosta et al., 2017). On the other hand, no significant differences were found in Peterson's study between imagined forward straight walking and turning in PD_FOG_ and PD_NOFOG_ (Peterson et al., 2014). The lack of significant difference might be attributed to the differences in the MI tasks used in their study, or the brain activation associated with their MI tasks was too subtle to produce detectable activity changes. In the current study, we incorporated the videos of different gait conditions to explicitly guide MI performance of participants, and it consistently elicited different brain responses to different gait conditions. This may be one of the pivotal reasons that cause the discrepancy between Peterson's study and the current study.

### Correlation Between Gait Parameters and FOG

In this study, PD_FOG_ had the slowest gait, smallest stride length, increased stance time, decreased swing time, least single support time, and increased total double support time. These results are in line with those of recently conducted studies (Vervoort et al., 2016; Mitchell et al., 2019). However, no significant correlation between gait parameters and brain activation was detected in this study. This suggested that FOG was a complex phenomenon involving multiple brain areas or brain networks. It could not simply be explained by a single gait parameter or the activity in one single brain region. A recent study illustrated that gait variability of stride length and walking velocity positively correlated with precuneus neural activity in PD_FOG_ (Bommarito et al., 2020). The finding charged a new possibility for exploiting the relationship among the sophisticated FOG brain activities and gait parameters in the future.

## Conclusions

In conclusion, the central neural function of PD_FOG_ differs from that of PD_NOFOG_. The alternation of the central neural function in PD_FOG_ can be triggered by the MI of various walking conditions. Complex gait requires more complex neural control than simple gait in both normal and FOG conditions, and the escalation of the locomotion region activities was required for compensating the insufficient function of fronto-striatal circuitry during the MI of complex gait in PD_FOG_ than in other participant groups.

## Data Availability Statement

The raw data supporting the conclusions of this article will be made available by the authors, without undue reservation.

## Ethics Statement

The studies involving human participants were reviewed and approved by China Medical University and Hospital Research Ethics Committee. The patients/participants provided their written informed consent to participate in this study.

## Author Contributions

B-LL, C-MC, H-CH, J-CC, M-KL, J-RD, and C-HT were responsible for the conception and design of the study. B-LL, C-MC, H-CH, C-IL, H-CL, and G-JW contributed to the collection and analysis of data. H-CH, M-KL, J-RD, and C-HT were responsible for the drafting of the manuscript. All authors critically revised the draft and approved the final version.

## Funding

This study was supported in part by grants from the Ministry of Science and Technology (MOST 105-2314-B-039-004-MY2, MOST 106-2410-H-008-054-, MOST 107-2314-B-039-017-MY3, MOST 107-2221-E-008-072-MY2, MOST 105-2410-H-039-003-, and MOST 110-2314-B-039-039-) and the China Medical University Hospital (Taiwan) (DMR-105-055, DMR-110-218, and DMR-109-069).

## Conflict of Interest

The authors declare that the research was conducted in the absence of any commercial or financial relationships that could be construed as a potential conflict of interest.

## Publisher's Note

All claims expressed in this article are solely those of the authors and do not necessarily represent those of their affiliated organizations, or those of the publisher, the editors and the reviewers. Any product that may be evaluated in this article, or claim that may be made by its manufacturer, is not guaranteed or endorsed by the publisher.
